# Impact of Vaping on Lungs: An Indian Prospect

**DOI:** 10.7759/cureus.48281

**Published:** 2023-11-04

**Authors:** Karan Sharma, Roshan K Jha

**Affiliations:** 1 Medicine, Jawaharlal Nehru Medical College, Datta Meghe Institute of Higher Education and Research, Wardha, IND; 2 Biochemistry, Jawaharlal Nehru Medical College, Datta Meghe Institute of Higher Education and Research, Wardha, IND

**Keywords:** e cigerette, e-cigarette or vaping product use associated lung injury (evali), e-cigarette smoking, vaping-induced lung injury, e-cigarette and vaping product use associated lung injury (evali)

## Abstract

Electronic cigarettes, or vape pens, have gained popularity among young people due to their attractive appearance, but they also have numerous side effects. These devices come in various shapes, sizes, and costs, with many brands and various flavors to choose from. As of now, there are around 2,807 people hospitalized in the United States due to vaping. Electronic cigarettes are illegal in many countries, with laws varying from country to country. The health service has been criticized for the implementation of the ban, with states implementing month-long drives and submitting reports. The Indian Police have also conducted raids and arrested five individuals under the 2019 ban on Electronic Cigarettes (Production, Manufacture, Trade, Transport, Sale, Distribution, Storage, and Advertisement) Act. The history of vapes can be traced back to the invention of the first electric vaporizer in 1927 by Joseph Robinson. Other pioneers like Herbert Gilbert and Jed Rose developed nicotine fixes using refined smoke. The vape was made in 2003 by Chinese smoker Hon Lik, who created the device as a better option to traditional smoking.

## Introduction and background

Nowadays, electronic cigarettes, or vape pens, are very popular among young people. It has now turned into a pattern to utilize a vape without a doubt even though it has numerous side effects that the youths don't realize. This little gadget comes in different shapes, sizes, costs, and limits. It goes from around 20$ can go up to 10,000$. There are over 450 brands and 7,700 flavors to choose from when vaping. There were approximately 2,807 people hospitalized from 50 states in the United States as of February 18, 2020 [1]. This device is illegal in numerous nations, and the laws vary from nation to nation. This is more harmful than smoking as numerous substances are present that can cause a ton of harm to the human body. A cigarette goes on for approximately 5 minutes yet vaping accompanies a great deal of puffs, some are likewise battery-powered and can be utilized for quite a while. Some of them can be refilled and utilized again. In India, in September 2019 the association bureau gave an ultimatum that restricted Electronic Nicotine Delivery Systems (ENDS), three months after this the service was boycotted. The concern is shared by educators, parents, and anti-vaping activists; two legal disputes recorded last year have made the well-being service observe the unfortunate implementation of the prohibition on e-cigarettes [2]. It has instructed states to carry out month-long drives to implement the ban and to submit reports detailing the actions taken. Additionally, it reached out to law enforcement personnel and held a national review meeting last month. In June 2022, the initial PIL was filed in Rajasthan [3]. A month later, under the 2019 Prohibition of Electronic Cigarettes (Production, Manufacture, Import, Export, Transport, Sale, Distribution, Storage, and Advertisement) Act, the Jaipur Police conducted raids and booked five individuals. The police chief additionally gave letters to the representatives to coordinate exceptional drives and missions to carry out the consequences and make a move against the wrongdoers. They are also developing strategies to monitor online sales of vaping products [4].

## Review

Methodology 

**Figure 1:**
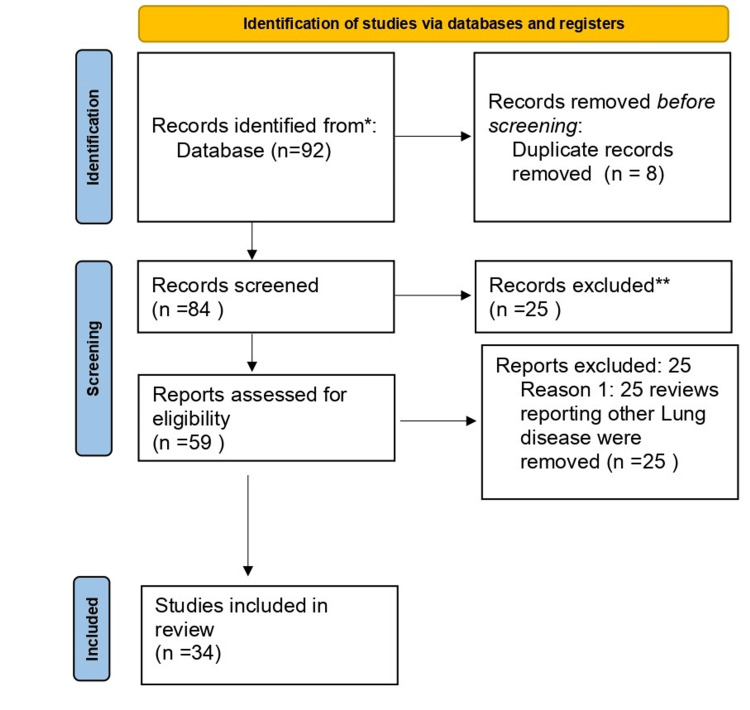
PRISMA flowchart for literature search PRISMA: Preferred Reporting Items for Systematic Reviews and Meta-Analyses

Using the electronic databases PubMed, Google Scholar, and ReasearchGate, a thorough search was done in English. It was also the subject of an independent search. Such search phrases as "EVALI," "Lung related injury," or "E-cigarette abuse. "The writers' expertise and familiarity with the subject helped them archive pertinent materials. This review includes articles that meet the following criteria: they must be written in English, they must be published within the last ten years, and they must be focused on vapes, nicotine addiction, and lung injury. Figure 1 (the Preferred Reporting Items for Systematic Reviews and Meta-Analyses (PRISMA) diagram) illustrates the research approach.

What is a vape?

Oral inhalation of nicotine-infused vapors is usually from a battery-operated electronic device that heats a solid or liquid. Testing of the aerosol sample found that there were approximately 150 chemicals, 104 of which were undetected, 25 of which were detected, 9 of which were too low to quantify, and 16 of which occurred entirely in the e-cigarette. In some samples, glycerol (average 37 g/100 g), propylene glycol (average 10 g/100 g), 1,3-propanediol, thujone, and ethyl vanillin were mainly detected. Daily users are estimated to consume 0.38 mg/kg/day of nicotine, 14.5 mg/kg/day of 1,2-propanediol, and 0.2 mg/kg/day of other compounds. Various synthetic flavors are mixed into these compounds like grapes, orange, kiwi, mango, pineapple, guava, jamun, etc. The mint flavor is very addictive and produces a lot of smoke as it contains methanol it is very similar in taste to mint it is highly addictive and harmful to the body in addition to mint flavor other flavors have an additional impact creating an illusion that it is soothing and not harmful to the body. Flavors are made to taste the same or better than the original product [4].

History of vapes 

For centuries people have shared the surge that tobacco gives. When Dr. Benjamin Surge pointed it out and alluded to smoking as "hostile," a long time back. He furthermore communicated that it can incite ailments and diseases. From that point, each trendsetter has been endeavoring to figure out how to fulfill nicotine needs without stinky cigarettes. Joseph Robinson had a vision for the primary electric vaporizer in 1927. He called this gadget "therapeutic compounds." Individuals kept on smoking, but not until the 1950s and 1960s did individuals begin to stress the well-being impacts of cigarettes [5]. In 1963 another man, Herbert Gilbert, initiated this "smokeless non-tobacco cigarette," in any case he was incapable of tracking down any creators to efficiently manufacture his item. Various others endeavored to create e-cigarettes [6]. Within the 1980s, Jed Rose made a nicotine fix utilizing "refined smoke. "The vape precursor was a long way to some degree radical. Rose said in 2000, a tabletop "spring of spouting magma vaporizer" entered the showcase not precisely expecting tobacco. The e-cigarette was made in 2003 by Chinese smoker Hon Lik, who cultivated the contraption to act as an alternative in differentiate to standard smoking. It is made out of a battery part, an atomizer, and a cartridge either a nicotine or non-nicotine liquid course of action. Around 2006 vaping was prevalent in Europe and it progressed towards the USA quickly. NJOY built up critical e-cigarette brands in 2007 in the USA [7].

Effects of vaping on lungs 

The use of electronic cigarettes creates fumes from a liquid that the person breathes in. The flavorings in the fluid may be the main attraction for young people to start smoking electronic cigarettes. It has been proven that inhaling the vapors from e-cigarettes causes a lot of harm [8]. This study demonstrates that distinct original studies on the hazardous nature of flavored The Scopus and PubMed databases were used to find and analyze e-liquids. Flavorings in flavored fluids or mist concentrates from e-cigarettes have caused harm to many people's skin, skeletal structure, cardiovascular and respiratory systems, and respiratory tracts [9]. Cinnamaldehyde, vanillin, menthol, ethyl maltol, ethyl vanillin, benzaldehyde, and linalool were the most frequently reported cytotoxic substances. Additionally, modern e-cigarettes may be adapted to exhale dry plant fiber or concentrated extracts of cannabis [10]. It is also possible to modify current e-cigarettes so that users can inhale dry plant fiber or a concentrated extract of cannabis. In 2019, there was a rise in lung ailments in the US referred to as e-cigarette, or vaping, item use-associated lung injury (EVALI). When it came to drug usage, 82% of 2,022 hospitalized patients who had access to information admitted to using a vaping product that contained delta-9-tetrahydrocannabinol as of January 14, 2020. Thirty-three studies on EVALI stood out in our literature. An authority on thickening and diluting cannabis-based products, vitamin E acetic acid derivation, has been connected to the EVALI incident in epidemiological and research facility investigations [11]. However, because the chemistry of e-liquids is so intricate, more than one ingredient, component, or warm breakdown item may be tested for toxicity [12]. More study is required, especially on e-cigarettes (generation, control environments, etc.), e-liquids (composition, bulk, or vaped frame), modeled frameworks (cell sort, culture type, and dosimetry measurements), organic observing, previously used exposures to nicotine and flavoring buildups, causal experts, and equipment of EVALI poisonous quality [13].

E-cigarettes caused associated lung damage (EVALI) 

The use of electronic cigarettes creates fumes from propylene glycol, vegetable glycerin or glycerol, other chemicals (including those used to create flavors), and, in some cases, water that the person breathes in. The flavorings in the fluid may be the main attraction for young people to start smoking electronic cigarettes [14]. This study demonstrates that distinct original studies on the hazardous nature of vapes. The Scopus and PubMed databases were used to find and analyze e-liquids. Flavorings in flavored fluids or mist concentrates from e-cigarettes have caused harm to people's skin, skeletal structure, cardiovascular and respiratory systems, and respiratory tracts [15]. Cinnamaldehyde, vanillin, menthol, ethyl maltol, ethyl vanillin, benzaldehyde, and linalool were the most frequently reported cytotoxic substances. Additionally, modern e-cigarettes may be adapted to exhale dry plant fiber or concentrated extracts of cannabis [16]. Modern e-cigarettes can also be modified to exhale cannabis as a concentrated extract or dried plant fiber. Beginning in 2019, the United States experienced an increase in lung injuries known as e-cigarette, or vaping, item use-associated lung damage (EVALI) [17]. As of January 14, 2020, 82% of 2,022 hospitalized patients with information on drug use acknowledged using a vaping product containing delta-9-tetrahydrocannabinol. In our literature, 33 papers on EVALI stood out [18]. A diluent and thickening expert in cannabis-based goods, vitamin E acetic acid derivation, has been connected to the EVALI incident in epidemiological and research facility investigations [19]. However, because the chemistry of e-liquids is so intricate, more than one ingredient, component, or warm breakdown item may be tested for toxicity [20]. More studies are required, especially on e-cigarettes (generation, control environments, etc.), e-liquids (composition, bulk, or vaped frame), modeled frameworks (cell sort, culture type, and dosimetry measurements), organic observing, previously used exposures to nicotine and flavoring buildups, causal experts, and equipment of EVALI poisonous quality [21].

Delta-8-Tetrahydrocannabinol (Δ8-THC)

It is the Δ9-THC double bond isomer. Two receptor subtypes, cannabinoid receptor types 1 and 2 (CB1 and CB2, respectively), are part of the human endocannabinoid system. Based on functional tests, the majority of research have found that Δ9-THC functions at both receptors as a partial agonist. The CB1 receptor is necessary for the psychoactive effects of Δ9-THC, according to clinical research including antagonists. Its lesser effects compared to many synthetic agonists with strong intrinsic activity can be explained by its comparatively low intrinsic activity. Although there are a few significant variations, the pharmacology of Δ8-THC and Δ9-THC in vitro, in vivo, and in humans is typically rather comparable. The primary distinction is that in several tests, Δ8-THC exhibits lower potency than Δ9-THC, while appearing to have comparable in vivo maximum effects and in vitro intrinsic effectiveness. Like Δ9-THC, Δ8-THC also has an active 11-OH metabolite that is more powerful than its parent. Their metabolisms are comparable. There are several possible explanations for the potential decreased in vivo potency of Δ8-THC, such as variations in potency at the CB1 receptor, variations in the ratio of CB1 to CB2 receptor activation, and variations in the rate of production of the active 11-OH metabolite [22].

Diseases caused due to vaping 

Diffuse Alveolar Hemorrhage (DAH)

DAH is a distinct pathophysiological illness marked by pulmonary bleeding that comes from the venules, arterioles, and alveolar capillaries that make up the pulmonary microcirculation. The clinical signs include hemoptysis, anemia, diffuse lung infiltration, and sudden respiratory failure. The diagnosis is confirmed by the presence of hemosiderin-loaded macrophages, fibrin, or red blood cells (RBCs) in the alveolar space on pathological findings. After 48-72 hours of bleeding, hemosiderin, a byproduct of hemoglobin degradation, appears and assists in differentiating DAH from surgical trauma. It is also possible to see widespread alveolar injury, organized pneumonia, or mild interstitial thickening [23].

Acute Eosinophilic Pneumonia (AEP)

This uncommon illness primarily affects men in their teens and early 30s. Even though the exact reason is frequently unknown, a direct correlation between smoking behaviors and triggering events has been consistently shown in connection to initiating, increasing, or resuming smoking after stopping. It is a fast-moving illness that frequently results in mortality and causes fever, dyspnea, and hypoxemic respiratory failure [24]. 

Pulmonary Langerhans' Cell Histiocytosis (PLCH)

Just 5% to 15% of patients with PLCH have multiorgan involvement, such as skin lesions, bone lesions, and diabetes insipidus. PLCH is limited to the lungs. Ninety percent or more of instances with PLCH are reported to be smokers; the condition is unusual and is detected in patients undergoing open lung biopsy for chronic interstitial disease. The pathological cause of PLCH is mostly unclear [25].

Harmful effects of nicotine

Disruption of nicotinic acetylcholine receptor (nAChR) with early nicotine use can also have an impact on the pharmacology of the receptor subunits and alter dopamine release, including glutamate, acetylcholine, dopamine, serotonin, and gamma-aminobutyric acid (GABA) [26]. In this review, we underscore that the effects of nicotine are contingent upon the time of exposure, including a dynamic interaction with reinforcement from dopaminergic, endocannabinoid, as well as opioidergic structures [27].In order to examine reviews in the literature, we searched the digital libraries of PubMed and Google Scholar for English-language publications published between January 1968 to November 2018 about teenage drug use, including vaping and the effects of nicotine on developing minds. [28]. Particularly in non-smoking clients, and also when used over an extended period. Because of the way such items are shipped, there might be dental health repercussions. There has been an increase in studies to determine the effects on oral fitness during the past several years. Numerous cell effects have been reported by in vitro studies, although these are vastly underrepresented compared to those caused by exposure to cigarette smoke [29]. According to microbiological studies, e-cigarette users have an altered microbiome, and there are some signs that this microbiome may be more pathogenic than that of non-users. There is yet little evidence that medicinal trials have an impact on oral fitness, and the majority of the research conducted so far has been small-scale and mainly cross-sectional [30,31].

Actions taken by countries

The anti-e-cigarette development is picking up more footing all over the world. India joined the list of countries that have successfully prohibited the deal, moment, publicizing, and generation of electronic cigarettes on September 18, 2019 [32]. A crisis law that has to be enacted by parliament has the support of Indian Prime Minister Narendra Modi. First-time offenders face a maximum sentence of one year in prison and a fine of 100,000 rupees (almost $1,400), while repeat offenders face three years in prison and a fine of 500,000 rupees (about $7,500) [33,34]. Additionally, owning e-cigarettes or similar devices will be illegal and punishable by up to a half-year jail sentence and a fine of up to 50,000 rupees (~$700) [35]. A crisis law that has to be enacted by parliament has the support of Indian Prime Minister Narendra Modi. First-time offenders face a maximum sentence of one year in prison and a fine of 100,000 rupees (almost $1,400), while repeat offenders face three years in prison and a fine of 500,000 rupees (about $7,500) [33,34]. Additionally, owning e-cigarettes or similar devices will be illegal and punishable by up to a half-year jail sentence and a fine of up to 50,000 rupees (~$700) [35]. While Japan permits the sale and distribution of "heat-not-consume" tobacco products and non-nicotine e-cigarettes, Singapore has a strong internal prohibition on e-cigarettes [36]. Due to severe regulations regarding fluid nicotine, vaping devices that use nicotine-containing e-juices are not permitted in Japan. Non-nicotine electronic cigarettes (e-cigarettes) and warmed smoking gadgets, such as "I Stopped Unique Smoking" (IQOS), are broadly conveyed [37].

## Conclusions

This is a very critical stage for stopping vapes as this is a very fast-growing market, and if not stopped timely this can lead to adverse consequences in which countries will have unhealthy and unwell youth population. Although using vapes has dire consequences, the youth have been using vapes without realizing it as they are always being advertised as better than smoking. There are many different brands of these gadgets on the market, and they come in a variety of forms, sizes, and tastes. However, the usage of vape pens has increased the frequency of hospitalizations, especially in the US, where thousands of individuals have been hospitalized due to diseases associated with vaping. Different nations have different bans on e-cigarettes, and many have rigorous laws in place to regulate their creation, sale, distribution, and promotion. Numerous nations have taken measures to address the dangers of e-cigarette use. For instance, India prohibited the manufacture, marketing, distribution, and sale of e-cigarettes in 2019. Other nations with strong laws governing the use of vaping devices include Singapore and Japan. Concerns about how vaping affects the lungs have been developing. EVALI, or severe vaping-related respiratory diseases, have become more common in recent years. Inhaling nicotine-laced vape support youngsters at the time can cause lung tissue damage and inflammation, which can cause symptoms including coughing, breathlessness, and chest discomfort. Flavored e-liquids include dangerous chemicals that can affect the respiratory system and have been connected to EVALI. This is not an addiction that is very hard to control it is rather easy if the parents or guardians support the youngsters while withdrawing the usage of vapes.
